# Aneurysmal Subarachnoid Hemorrhage and Neuroinflammation: A Comprehensive Review

**DOI:** 10.3390/ijms17040497

**Published:** 2016-04-02

**Authors:** Brandon P. Lucke-Wold, Aric F. Logsdon, Branavan Manoranjan, Ryan C. Turner, Evan McConnell, George Edward Vates, Jason D. Huber, Charles L. Rosen, J. Marc Simard

**Affiliations:** 1 Department of Neurosurgery, West Virginia University School of Medicine, Morgantown, WV 26505, USA; Bwold@mix.wvu.edu (B.P.L.-W.); rcturner@hsc.wvu.edu (R.C.T.); crosen@hsc.wvu.edu (C.L.R.); 2 Department of Basic Pharmaceutical Sciences, West Virginia University School of Pharmacy, Morgantown, WV 26505, USA; logsdoa@gmail.com (A.F.L.); jdhuber@hsc.wvu.edu (J.D.H.); 3 McMaster Stem Cell and Cancer Research Institute, Michael G. DeGroote School of Medicine, Hamilton, ON L8S 4K1, Canada; branavan.manoranjan@medportal.ca; 4 Department of Neurobiology and Anatomy, University of Rochester Medical Center, Rochester, NY 14642, USA; evan_mcconnell@urmc.rochester.edu (E.M.); edward_vates@urmc.rochester.edu (G.E.V.); 5 Departments of Neurosurgery, Pathology, and Physiology, University of Maryland School of Medicine, Baltimore, MD 21201, USA

**Keywords:** aneurysmal subarachnoid hemorrhage, cerebral vasospasm, neuroinflammation, novel treatments

## Abstract

Aneurysmal subarachnoid hemorrhage (SAH) can lead to devastating outcomes including vasospasm, cognitive decline, and even death. Currently, treatment options are limited for this potentially life threatening injury. Recent evidence suggests that neuroinflammation plays a critical role in injury expansion and brain damage. Red blood cell breakdown products can lead to the release of inflammatory cytokines that trigger vasospasm and tissue injury. Preclinical models have been used successfully to improve understanding about neuroinflammation following aneurysmal rupture. The focus of this review is to provide an overview of how neuroinflammation relates to secondary outcomes such as vasospasm after aneurysmal rupture and to critically discuss pharmaceutical agents that warrant further investigation for the treatment of subarachnoid hemorrhage. We provide a concise overview of the neuroinflammatory pathways that are upregulated following aneurysmal rupture and how these pathways correlate to long-term outcomes. Treatment of aneurysm rupture is limited and few pharmaceutical drugs are available. Through improved understanding of biochemical mechanisms of injury, novel treatment solutions are being developed that target neuroinflammation. In the final sections of this review, we highlight a few of these novel treatment approaches and emphasize why targeting neuroinflammation following aneurysmal subarachnoid hemorrhage may improve patient care. We encourage ongoing research into the pathophysiology of aneurysmal subarachnoid hemorrhage, especially in regards to neuroinflammatory cascades and the translation to randomized clinical trials.

## 1. Introduction

Aneurysmal subarachnoid hemorrhage (SAH) can lead to devastating outcomes for patients, including cognitive decline, cerebral vasospasm (CV), and delayed cerebral ischemia [1,2]. The underlying mechanisms contributing to injury expansion following aneurysmal SAH are poorly understood, therefore limiting the number of effective pharmaceutical treatment options. Recent evidence implicates neuroinflammation as a key mediator of injury expansion and behavioral deficits [3,4]. Peripheral immune cells are both recruited and activated in damaged tissue [5]. These cells can enter the brain parenchyma and release inflammatory cytokines [6]. Additionally, intrinsic toll-like receptors are upregulated after infarction leading to widespread neuroinflammation [7]. Furthermore, neuroinflammation has been linked to adverse secondary outcomes that occur after SAH. Vessels undergoing CV have increased leukocyte adhesion capacity contributing to delayed neurologic deterioration [8,9]. In this review, we highlight what is known about neuroinflammation following aneurysmal SAH. We discuss how neuroinflammation contributes to CV and delayed cerebral ischemia, how neuroinflammation susceptibility is affected by comorbidities and genetics, and the potential benefit for targeting inflammatory pathways following aneurysmal SAH. Finally, we highlight potential avenues for future study including novel treatment approaches. Articles selected for this review were based on impact factor of journal, number of overall citations, and the general impact of the article for advancing understanding of SAH mechanisms.

## 2. Neuroinflammation

### 2.1. The Role of Inflammation

Emerging evidence points to inflammation playing a major role in acute and chronic phases of neural injury associated with aneurysmal SAH [10,11]. In the following sections, we review and explore some of the pathophysiology associated with aneursymal SAH, particularly in relation to the neuroinflammatory response. We conclude the section by highlighting new avenues and suggest further studies to address lingering questions within the field of aneurysmal SAH.

### 2.2. SAH Pathophysiology: Acute Events

The initial aneurysmal rupture deposits blood within the subarachnoid space. Red blood cell breakdown and degradation over time leads to the deposition of hemoglobin. Methemoglobin, heme, and hemin resulting from red blood cell breakdown can lead to activation of TLR4, which signals inflammatory cascades that damage neurons and white matter [12,13]. Hemin has been linked with the release of redox-active iron, altering the balance of oxidants and anti-oxidants. The redox-active iron depletes anti-oxidant stores such as nicotinamide adenine dinucleotide phosphate (NADPH) and glutathione while producing superoxide and hydroxyl radicals as well as lipid peroxidation [14,15].

As blood leaves the confines of the vasculature following aneurysmal rupture, immunomodulatory cells within the CNS, such as microglia, are activated. These cells trigger the upregulation of numerous cell adhesion molecules within endothelial cells, which subsequently allows a multitude of inflammatory cells to bind and enter the subarachnoid space [16,17]. Once in the subarachnoid space, these inflammatory cells, macrophages and neutrophils, phagocytize the extravasated, degrading red blood cells [12]. This process occurs in an effort to clear free hemoglobin, and promote neurostability and recovery. Hemoglobin clearance is facilitated by the binding of hemoglobin to haptoglobin for rapid engulfment by immune cells [13].

### 2.3. SAH Pathophysiology: Subacute-Chronic Events

As described above, pre-clinical data suggest that aneurysmal SAH is associated with the attraction and subsequent deposition of neutrophils and macrophages in response to free hemoglobin and hemin. A key area requiring further investigation is how these neutrophils and macrophages are recruited and whether they pass through an intact or disrupted blood brain barrier. While these peripheral immune cells are essential for clearing hemoglobin, the cells can become trapped in the subarachnoid space due to alterations in cerebral spinal fluid (CSF) flow and the restoration of the endothelial tight junction barrier. Once trapped within the subarachnoid space, the macrophages and neutrophils undergo degranulation, which releases a multitude of inflammatory factors. A few of these include endothelins and oxidative radicals. These factors can cause inflammation-induced vasoconstriction, arterial narrowing, meningitis, and cerebritis [18]. Importantly, the inflammatory response is generalized, causing the release of inflammatory cytokines, endothelial adhesion molecules, and activated complement throughout the brain [19,20].

### 2.4. Inflammatory Mediators in SAH: A Focus on Cytokines and Cell Lines

Inflammation following aneursymal SAH has been investigated in order to characterize the timing, magnitude, and site of cytokine release [21,22]. For example, IL-1β, IL-6, and TNFα are released into both the serum and cerebrospinal fluid following SAH [23,24]. Similarly, these same cytokines have been shown in animal models of SAH to be increased in the cerebral arterial wall [25]. While the role that these cytokines play in SAH warrants further investigation, prevailing clinical signs such as neutrophilia, pyrexia, and general cerebral edema are likely associated with the cytokine storm [26]. The current lack of clarity with regards to the beneficial or detrimental role inflammation plays following aneurysmal SAH is perhaps best exemplified by the mixed results of preclinical and clinical studies. Some clinical studies have found that modulating inflammation following SAH is beneficial while other studies have shown no beneficial effect at all [27]. It is likely that activation of inflammation at different time points post rupture is associated with different protective or detrimental responses depending on the surrounding milieu and type of cells recruited to the site.

What is apparent is the prominent role inflammation plays in causing cerebral vasospasm (CV). Injection of pro-inflammatory materials/compounds intracisternally induces CV, even in the absence of blood breakdown products [1]. Clinically, the inflammatory response appears in close temporal relationship with the spasm and in direct proportion to the magnitude of the inflammatory response [28]. These findings have been supported by evidence suggesting that accumulation of inflammatory cells closely parallels neuronal cell death. Cell death near the vasculature has been substantially reduced by depletion of inflammatory cells in preclinical studies [29].

A substantial knowledge gap persists in understanding the adaptive immune response in SAH. Emerging evidence from preclinical studies has implicated B and T lymphocyte infiltration into the vessel wall of aneurysms, which may potentially promote aneurysmal rupture and SAH [30,31]. Preliminary data from clinical studies indicate a diffuse presence of not only innate immune cells but also adaptive immune cell populations in the CSF and serum following SAH [5]. While it is difficult to draw conclusions based upon this preliminary study, it is promising in that a new therapeutic target and avenue may be available in the future [32,33]. CSF biomarkers may be used to guide physicians on the appropriate selection of pharmaceutical agents.

### 2.5. Inflammatory Mediators in SAH: A Focus on Proteases

Additional inflammation-specific SAH studies have focused on matrix metalloproteinases (MMPs), a family of proteases consisting of multiple subtypes. The most widely investigated of these being MMP-9 [34,35]. MMP-9 has been shown to be responsible for the degradation of tight junction proteins, which are critical in the maintenance of blood-brain barrier (BBB) integrity. Notably, clinical studies of SAH have reported an elevation of MMP-9 in brain tissue, serum, and cerebrospinal fluid [36,37]. Consistent with cytokine studies, MMP-9 is elevated not only in serum and CSF but also in the vessel wall [25]. These studies document a potential novel approach for treatment of SAH. By targeting MMP-9 in the vessel wall, it could be possible to restore BBB integrity or prevent BBB disruption from occurring in the first place. Preventing basement membrane degradation could facilitate enhanced reconstitution of tight junction protein binding.

### 2.6. SAH-Associated Inflammation: An Inflow or an Outflow Problem?

CSF flow disruption is associated with inflammation following SAH [38]. This is consistent with clinical symptoms where obstructive hydrocephalus is commonly reported due to the presumed breakdown of blood products in the subarachnoid space and subsequent obstruction of CSF drainage pathways. The etiology of this condition is actually poorly understood and may in fact be due to obstruction of the glymphatic system described by Nedergaard and colleagues [39]. The system is critical for CSF circulation and consists of multiple channels around the vasculature. While the glymphatic system remains under investigation in several neurological injury models such as traumatic brain injury, what has already been elucidated is that the system plays a critical role in elimination of metabolic waste products both during normal homeostasis and after injury. The glymphatic system functionality can be altered in the context of neural injury, such as traumatic brain injury, as well as in sleep deprivation [40]. Ongoing preclinical studies are being conducted to determine if the glymphatic system is disrupted following aneurysmal SAH. We expect SAH to cause a significant disruption in glymphatic flow. How this knowledge can be utilized for enhancing treatment after SAH is unclear but will likely be of value clinically and therapeutically. Agents that can restore glymphatic flow will be of value clinically.

Another challenge with regards to modulating inflammation is the fact that inflammation is often observed to be biphasic in nature, with elements that are both protective as well as deleterious. Identifying this temporal relationship and when to target involved pathways for therapeutic benefit remains a substantial challenge. The magnitude of the inflammatory response may dictate outcome, and warrants further investigation with preclinical models. Advanced neuroimaging may offer a viable option to detect biphasic peaks in the neuroinflammatory cascade. Finally, utilizing current knowledge regarding SAH pathophysiology offers clear advantages therapeutically. For example, patients with the haptoglobin α1–α1 subunit compared to the haptoglobin α2–α2 subunit have decreased risk of CV following aneurysmal SAH, indicating a potential therapeutic target [41].

## 3. Secondary Outcomes

### 3.1. Cerebral Vasospasm

Aneurysmal SAH can lead to CV, which is the narrowing of blood vessels within the subarachnoid space of the brain [42]. It is a serious complication often experienced by aneurysmal SAH survivors [43,44]. If CV persists, insufficient blood flow reaches affected regions of the brain, causing delayed cerebral ischemia [45]. CV is characterized by progressive narrowing of cerebral arteries beginning no earlier than day three following hemorrhage and peaking at one week [46]. Clinically, CV leads to delayed cerebral ischemia and infarction in 20%–30% of patients [47]. While the etiology of CV remains unknown, spasmogenic and neuroinflammatory substances generated from the lysis of subarachnoid blood are thought to drive the process [48]. Given that few treatments effectively target CV, novel murine models have been developed to further elucidate the mechanisms that regulate CV. Specifically, models have been designed to simulate vasodilatory mechanisms of the cerebral vasculature [49,50,51,52]. One mouse model involves endovascular perforation of the middle cerebral artery to produce the hemorrhage [51,52]. Another CV model consists of injecting femoral arterial blood intradurally through the atlanto-occipital membrane [50]. A canine model has been established with double hemorrhage that reliably produces CV [53]. Although all the models generate vascular changes consistent with CV, the endovascular perforation model is much more lethal, with a mortality of 29% [51,52], compared to the 3% in the intradural injection model [50].

### 3.2. CV and Inflammation

Growing evidence supports a role for neuroinflammation in the pathogenesis of CV [48,54]. Preclinical models of CV have repeatedly demonstrated the presence of cytokine upregulation and neutrophil activation. Active neutrophils can promote reactive oxygen species formation, which has been implicated in vascular pathology [55]. Further work needs to be done in order to elucidate the mechanisms by which these neutrophils are recruited to the site of rupture. In human clinical studies, the cerebrospinal fluid (CSF) neutrophil percentage is an independent predictor of CV in SAH patients [55]. Specifically, a CSF neutrophil content of >62% on day three following a SAH serves as an independent predictor for developing CV [55]. Therefore, the resulting neutrophil-mediated inflammatory process offers a potential therapeutic window for the treatment and prevention of CV.

**Figure 1:**
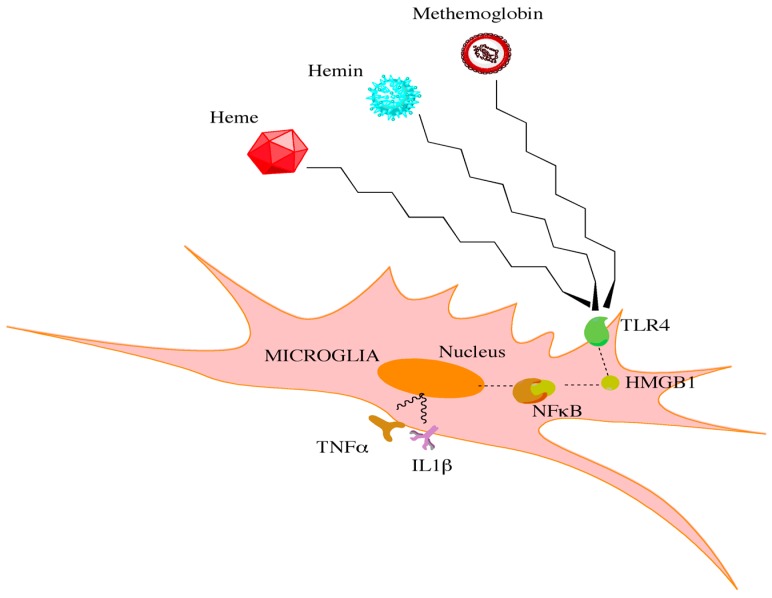
Red blood cell breakdown causes the release of heme, hemin, and methemoglobin. Through interactions with toll-like receptors on microglia, high mobility group box 1 protein is increased. This increase leads to downstream activation of NFκB and the release of proinflammatory cytokines.

While specific cell types may have unique roles in initiating CV, shared signaling pathways within these distinct populations may converge on common downstream effectors to drive CV. Heme released from hemoglobin has been shown in a preclinical model to promote a significant neuroinflammatory response, in part through signaling via toll-like receptor 4 (TLR4) [56,57]. TLR4 subsequently interacts with downstream effectors to generate a bimodal nuclear factor kappa beta (NFκB)-dependent inflammatory response (Figure 1) [58,59,60]. TLR4^−/−^ mice demonstrate a significant reduction in CV following SAH when compared to wild-type mice. Interestingly, TLR4 agonists alter the degree of CV in TLR4^−/−^ mice such that it mimics wild-type mice [58]. Furthermore, attenuation of the TLR4 and NFκB-dependent inflammatory response reduced the expression of downstream pro-inflammatory factors and provided a neuroprotective role in an aneurysmal SAH model [61]. These studies further establish TLR4 as a critical component in the CV cascade. Although TLR4 is expressed across many cell types, it is mostly expressed in microglia during the bimodal phases of CV [58]. Depletion of microglia *in vivo* following SAH is able to significantly reduce the extent of CV during both phases of the cascade [58]. However, the limited clinical utility in depleting microglia is apparent. Therapeutics that can dampen the microglia response may be beneficial however in reducing this inflammatory cascade.

### 3.3. CV and Long-Term Deficits

Whereas CV represents an acute concern in SAH patients, lasting cognitive deficits are a common long-term complication observed in aneurysmal SAH survivors [62]. SAH survivors also experience functional impairments in their ability to perform daily activities [62]. An assessment of grey- and white-matter damage following aneurysmal SAH in rats demonstrated a significant decrease in the neuronal marker microtubule-associated protein 2 and myelin basic protein, respectively [63]. Glial fibrillary acidic protein (GFAP), a marker of astrocytes, was increased in rat brains following SAH suggesting the presence of reactive gliosis [63]. Activated astrocytes can form glial scars, which are protective in the acute phase of brain injury by establishing a clear boundary between damaged and healthy tissue and thereby preventing the spread of pro-inflammatory signals throughout the brain [64,65]. However, ongoing reactive gliosis and scar formation may inhibit axonal regrowth and remyelination, further promoting grey- and white-matter damage in response to a persistent inflammatory response [66]. Targeting the chronic but not acute gliosis may be a viable treatment option worth further study.

The effects of aneursymal SAH can also result in acute and long-term functional deficits in sensorimotor behavior [63]. Both mechanical sensitivity to innocuous stimuli and fine sensorimotor function were significantly impaired in SAH-induced rats when compared to sham-operated controls [63]. Therefore, it appears that the cognitive and functional deficits observed in SAH preclinical models and patients are primarily a consequence of the persistent neuroinflammatory process observed in these patients during the acute and later stages of recovery.

## 4. Etiology and Comorbidities

### 4.1. Genetic Factors

Age is a determining factor of outcome following aneurysmal SAH [67]. Aged vessels are less compliant and have weak muscular walls. Premature vascular aging contributes to an increased risk of aneurysmal rupture and subsequent hemorrhage. Patients pre-disposed to epoxyeicosatrienoic acid upregulation are more at risk for vascular dysfunction and aneurysmal rupture [68,69]. Mutations in *VCAN* gene have also been linked to SAH [70]. Neuroinflammation severely damages already dysfunctional vessels. After rupture has occurred, mutations in the 9p21 locus on gene *CDKN2A* increases neuroinflammation [71]. Interleukin 6 polymorphisms can also augment neuroinflammation and can worsen SAH outcomes [72]. Interestingly, patients with the haptoglobin phenotype Hp2-2 have increased susceptibility for CV post-rupture [73], which, as discussed above, may be mediated by neuroinflammation.

The apolipoprotein-ε4 allele has been associated with poor cognitive performance several years following aneursymal SAH in patients [74]. Certain polymorphisms of the *A1166C* gene, which regulates angiotensin II type 1 receptors, have also been correlated to worsened outcome after aneurysmal SAH [75]. These genetic associations are poorly understood in the clinical population. Pre-clinical genetic studies however have shed light on the role that neuroinflammation plays in injury progression.

In a rat SAH model, high-mobility group box 1 triggers NFκB translocation thereby promoting neuroinflammation (Figure 1) [76]. The subsequent release of TNFα severely compromises vascular integrity around the site of SAH [77]. Recently, Chen and colleagues discovered that the P2X7R/cryopyrosin inflammasome axis is genetically primed in certain rodent strains, which contributes to a surge in interleukin 1β (IL-1β) following aneurysmal SAH [78]. Future clinical studies are needed to verify how these pathways are altered in patients with SAH. The sulfonylurea receptor 1-transient receptor potential melastatin 4 (Sur1-TRPM4) channel was found to be transcriptionally upregulated by *Abcc8* and *Trpm4* in rodents and humans following SAH [79]. This phylogenetically conserved phenomenon makes it an ideal target for pharmacologic intervention, which we will discuss further in the concluding sections.

### 4.2. Comorbidities and Physical Correlates

Patients with underlying comorbidities often fare worse following aneursymal SAH. Patients with migraine headaches are at increased risk for SAH [80]. Diabetes mellitus type II is associated with an increased risk for CV following SAH [81]. Patients with hypertension have larger subdural clot volumes following SAH [82]. Only recently have investigators begun to look at the mechanistic links between these comorbidities and outcome.

**Figure 2:**
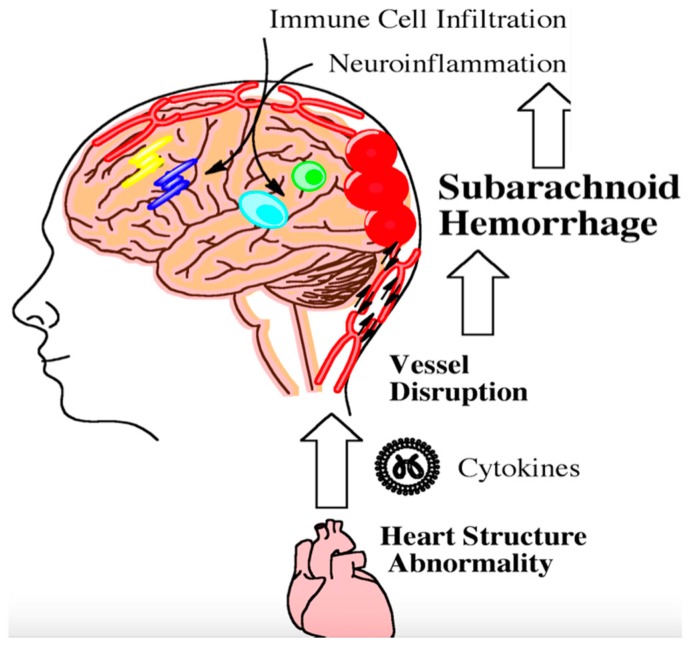
Following aneursym rupture, the brain stem can become ischemic and trigger heart damage. Damaged heart tissue can subsequently contribute to the inflammatory milieu following subarachnoid hemorrhage. Inflammation from heart abnormalities triggers the infiltration of peripheral immune cells into the brain as indicated by the arrows. This step-wise process further exacerbates neuroinflammation. Persistent neuroinflammation can lead to long-term cognitive and behavioral deficits.

The heart and brain are intimately connected through the vasculature and nerves. Once the brain becomes damaged from aneurysmal SAH, parasympathetic dysfunction may contribute to cardiac arrhythmias, leading to the release of inflammatory cytokines that enter the vasculature [83]. Modified vascular markers released following aneurysm rupture can interact with cytokines from damaged heart tissue and have been shown in a preclinical model to perpetuate inflammation within the brain (Figure 2) [84]. Preclinical data have shown that activated neutrophils in the peripheral vasculature can likewise damage brain microvessels following SAH [85]. Leukocyte diapedesis can occur through damaged microvessels following injury. Many peripheral immune cells migrate into the cerebral spinal fluid and the brain [86].

Inflammatory factors released from the damaged heart can perpetuate inflammation in the brain by means of an inflammatory circuit [83]. When comorbidities are present, this inflammatory circuit is accelerated [87]. Adipokines from excess fat storage can increase neuroinflammation susceptibility after brain injury [88]. Evidence suggests that atherosclerosis and diabetes can prime the immune system and exacerbate an inflammatory response during SAH [89]. Further investigation with clinical studies is needed to elucidate additional factors contributing to neuroinflammation in aneursymal SAH patients with comorbidities and/or the metabolic syndrome.

## 5. Targeting Neuroinflammation

### 5.1. Treatment

SAH can lead to devastating outcomes such as cognitive decline, CV, and delayed cerebral ischemia. The most common cause of SAH and the focus of this review is cerebral aneurysm rupture. The prevalence of cognitive decline and CV is high in surviving aneurysmal SAH patients. Patients who survive SAH surgery often display functional deficits in addition to long-term memory deficits [90]. SAH symptoms have proven difficult to prevent; however, an effective treatment algorithm can improve outcome and increase quality of life for surviving SAH patients.

The initial treatment for SAH after cerebral aneurysm rupture is to regulate and control extremes of blood pressure. Captopril and losartan act on the renin-angiotensin system and have been shown to reduce hemorrhage post-rupture and regulate blood pressure [91]. Nimodipine is a calcium channel blocker that has been shown to reduce poor outcome post-rupture [92]. Another drug that has been employed clinically to target neuroinflammation is an IL-1 receptor antagonist, which has been shown to reduce inflammatory cytokine levels within the CSF of patients with aneurysmal SAH [93]. TNFα inhibitors are also currently under investigation and warrant further consideration [4]. In order to improve available treatments, it is imperative that investigative work continues in pre-clinical models and that novel approaches for reducing neuroinflammation be developed.

### 5.2. Lessons from Animal Models

SAH models have produced similar deficits to those seen in patients following aneurysm rupture [61,63,94,95]. CV followed by delayed cerebral ischemia is a detrimental physiological outcome seen in patients with SAH. In a rodent model of SAH, vasoconstrictive receptor upregulation provided evidence of CV followed by delayed cerebral ischemia [96]. A recent study proposed that the MEK1/2 pathway regulates multiple contractile receptors and may be a viable target for reducing delayed ischemia after aneurysmal SAH [97].

Inflammation following delayed cerebral ischemia has been linked clinically to memory and functional deficits in SAH patients as discussed above. In light of this, many studies have focused on understanding inflammatory cascade activation in preclinical models of SAH (Table 1). Recently, groups have begun to investigate neuroprotective agents that may mitigate these inflammatory cascades. Maddahi and colleagues showed that mitogen activated protein kinase (MAPK) pathway inhibition reduced inflammation in a rodent model of SAH [98]. Previous work in spinal cord injury [99], intracerebral hemorrhage [100], brain ischemia [101], and hypoxic-ischemic brain injury [102] have implicated a neuroprotective role for the breast cancer chemotherapeutic agent, tamoxifen. Tamoxifen administration in a rat SAH model decreased inflammation, and the rats demonstrated no evidence of early brain damage such as cortical edema and BBB disruption [61]. Most strikingly, tamoxifen-treated rats had complete reversal of their SAH-induced spatial working memory dysfunction compared to vehicle-treated controls [61].

Peripheral immune cell adhesion and infiltration is yet another common secondary effect following SAH. This finding was elucidated first in rodent SAH models. The drug, LJP-1586, was shown to block vascular adhesion protein 1 and reduce leukocyte trafficking after SAH [103]. LJP-1586 provided improved cognitive and functional performance after SAH. Moreover, the immunomodulator, fingolimod, reduced leukocyte adhesion and improved neurological outcome in the EP model of SAH [104]. Preventing the infiltration of peripheral immune cells into the brain can reduce inflammation and improve neurological outcome following aneurysmal SAH. These early studies indicate that targeting neuroinflammation following SAH could be a viable therapeutic option for patients, and warrants further investigation in randomized clinical trials.

Treatment of CV is also the subject of ongoing studies. Potential therapeutic targets include E-selectin [105], a trafficking molecule for neutrophils and other inflammatory cells across the vascular endothelium, and CD11/CD18 [106], an adhesion molecule for neutrophils and macrophages. Preclinical therapeutics that block E-selectin [105] or antibodies administered against CD11/CD18 [106] have demonstrated a dramatic reduction in the severity of CV. These findings provide a proof-of-concept for treating CV by targeting neutrophils and preventing immune infiltration.

Additional therapies that target the myeloid lineage may elucidate a new role for these cells in the development of CV [107]. Ly6G/C is a cell surface marker unique to the myeloid lineage and primarily found on neutrophils and monocytes with some expression on CD8+ T-cells [108]. Myeloid cell depletion using an anti-Ly6G/C antibody prior to experimental SAH completely diminished angiographic CV as measured by middle cerebral artery diameter, and resulted in improved behavioral tests [107]. However, the clinical utility of depleting myeloid cells in intensive care patients is confounded by the increased risk of infection.

### 5.3. Novel Discoveries

Treatment options typically used for peripheral diseases are now being investigated for reducing the secondary effects of aneurysmal SAH. The anticoagulant, heparin, reduced inflammation and apoptosis in a rat model of SAH [109]. Tosun and colleagues investigated neuroinflammation and found the important involvement of Sur1-Trpm4 [79]. This receptor can be selectively inhibited with glibenclamide (glyburide), which was shown to reduce inflammation and behavioral deficits in a rodent model of SAH [79]. Glibenclamide is also a viable treatment option for ischemic and hemorrhagic stroke [3], and is now currently in phase II clinical trials for acute CNS injury [110].

To expand these findings, Dumont and colleagues proposed that CV may be mediated by inflammatory cytokines after SAH [111]. This idea is supported by the detection of vascular adhesion molecules in serum and CSF of SAH patients. The vascular molecules trigger a robust release of inflammatory cytokines [112]. Antioxidant therapies in rodent SAH models have shown promising results for reducing inflammation-mediated CV. The antioxidant rosiglitazone reduced CV and improved neurological outcome in both a rodent [113], and a rabbit model of SAH [114]. Additionally, glutamate toxicity has been shown to play an important role in the neuroinflammatory cascade and injury expansion following SAH [115]. Glutamate modulation was shown to reduce signs of CV in both human endothelial cells and in a mouse model of SAH [116].

## 6. Conclusions

Aneurysmal SAH continues to be a difficult clinical paradigm to treat with limited pharmacologic agents. New research has emerged that requires a careful re-examination of the role of neuroinflammation in CV, subsequent delayed cerebral ischemia, and overall patient outcome. Hemoglobin breaks down within the subarachnoid space and can trigger a robust inflammatory response. This response is coupled with an influx of peripheral immune cells and activation of innate immune cells within the brain. Additionally, genetic predisposition and associated comorbidities show that neuroinflammation itself might play a role in generating aneurysmal SAH. Novel treatment options targeting these neuroinflammatory cascades have proved efficacious in pre-clinical models for preventing CV, reducing delayed ischemia, and limiting long-term cognitive deficits (Table 1). These treatments should be rigorously evaluated in randomized double-blinded clinical trials. It is imperative going forward that research regarding the pathophysiology of SAH as it relates to neuroinflammation coincides with the search for drug discovery. This double-edged sword married with the appropriate preclinical models offers the best option for ultimate clinical success. Facilitating the translation of novel drugs from pre-clinical studies to clinical trials is essential for improving treatment options for patients with SAH.

## Figures and Tables

**Table 1:** Preclinical and clinical models of Subarachnoid Hemorrhage treatment.

Study	Sex/Species/Age	Model	Drug	Target	Outcome Measures
(a) Common cisternal SAH model					
Polvsen & Edvinsson 2015	Male SD Rats 2–3 months	Cisternal blood infusion	U0126	MEK1/2	Neurological score; Behavioral deficits; Cerebral blood flow; Endothelin receptor
Maddahi *et al.*, 2012	Male SD Rats 2–3 months	Cisternal blood infusion	U0126	MEK1/2	Neurological score; MAPK pathway; Pro-inflammatory activity; Matrix Metalloproteinase
Zhang *et al.*, 2014	Male SD Rats 2–3 months	Cisternal blood infusion	Astaxanthin	General Anti-oxidant Anti-inflammatory	Neurological score; Blood-brain barrier permeability; Edema; Pro-inflammatory activity; Leukocyte activity; Neuronal cell death
Pradilla *et al.*, 2004	New Zealand White Rabbit 1.5–2.5 kg	Cisternal blood infusion	Antibody	CD11/CD18	Blood vessel diameter (Vasospasm); Leukocyte activity
Provencio *et al.*, 2011	Male C57 Mice 2–3 months	Cisternal blood infusion	Antibody	Lymphocyte antigen 6 complex locus G6D (Myeloid cells)	Blood vessel diameter (Vasospasm); Leukocyte activity; Behavioral deficits; Microglial response
Lin *et al.*, 2005	Male C57 Mice 30–35 g	Cisternal blood infusion	Antibody	E-Selectin	Blood vessel diameter (Vasospasm); Leukocyte activity
Wu *et al.*, 2011	Male SD Rats 300–350 g	Cisternal blood infusion	Rosiglitazone	Peroxisome proliferator-activated receptor gamma agonist	Blood vessel diameter (Vasospasm); Leukocyte activity; Pro-inflammatory activity
Guresir *et al.*, 2013	Male SD Rats 250–350 g	Cisternal blood infusion	human recombinant Erythropoietin	Erythropoietin receptor	Neurological score; Blood vessel diameter (Vasospasm); Neuronal cell death
Germano *et al.*, 2007	Male SD Rats 250 g	Cisternal blood infusion	Felbamate	*N*-methyl-d-aspartate receptor antagonist	Blood-brain barrier permeability; Behavioral deficits; Body weight
Garzon-Muvdi *et al.*, 2013	C57 Mice 22–30 g	Cisternal blood infusion	S-4-carboxy-phenylglycine	Glutamate receptor antagonist	Blood vessel diameter (Vasospasm); Leukocyte activity;
(b) Clinically-relevant SAH models					
Xu *et al.*, 2015	Male SD Rats 2–3 months	Endovascular puncture	LJP-1586	Semicarbazide-sensitive amine oxidase inhibitor	Neurological score; Leukocyte activity; Microvascular damage
Xu *et al.*, 2015	Male SD Rats 2–3 months	Endovascular puncture	Fingolimod	Sphingosine-1-phosphate receptor modulator	Neurological score; Leukocyte activity; Microvascular damage
Simard *et al.*, 2012	Male Wistar Rats 300–350 g	Entorhinal cortex blood infusion	Heparin	Antithrombin III activator	Demyelination; Neurodegeneration; Pro-inflammatory activity
Tosun *et al.*, 2013	Male Wistar Rats 300–350 g	Entorhinal cortex blood infusion	Glibenclamide	Sur1-Trpm4 channel inhibitor	Neurodegeneration; Behavioral deficits
Makino *et al.*, 2012	Male C57 Mice 2–3 months	Induced hypertension + Elastase injection	Tetracycline Derivatives	Inflammatory Cytokines	Neurological score; Aneurysm rupture at 6 days
(c) Clinical Trials for SAH					
Singh *et al.*, 2014	Human	Clinical Trials	Anakinra	Interleukin-1 receptor anatagonist	Glasgow outcome score; Blood plasma and cerebral spinal fluid levels of Interleukin-6 between 6 and 24 h
Ma *et al.*, 2012	Human	Clinical Trials	Clazosentan	Endothelin receptor antagonist	Glasgow coma score; Post-SAH vasospasm; Late cerebral ischemia
Springborg *et al.*, 2007	Human	Clinical Trials	Erythropoietin	Erythropoietin receptor	Glasgow outcome score; Transcranial Doppler flow velocity; vasospasm; jugular venous oximetry; Brain injury markers; Blood-brain barrier integrity
